# Prevalence of Adverse Effects Among Prostate Cancer Patients Following Robotic-Assisted Laparoscopic Prostatectomy, External Beam Radiation Therapy, or Combined Therapy in North Florida

**DOI:** 10.7759/cureus.108781

**Published:** 2026-05-13

**Authors:** Nicole Fatovic, Juan Varela, Itamar Gal, Farigol Hakem Zadeh, Nikita Shah, Robert Carey, Victoria Bird

**Affiliations:** 1 Department of Applied Physiology and Kinesiology, University of Florida, Gainesville, USA; 2 Department of Urology, National Medical Association and Research Group, Gainesville, USA; 3 Department of Urology, University of Florida College of Medicine, Gainesville, USA; 4 Department of Biology, University of Florida, Gainesville, USA; 5 Department of Internal Medicine, University of Central Florida, HCA Florida North Florida Hospital, Gainesville, USA; 6 Department of Urology, Florida State University College of Medicine, Tallahassee, USA; 7 Department of Urology, HCA Florida North Florida Hospital, Gainesville, USA

**Keywords:** adverse effects, external beam radiation therapy, prostate cancer, radical prostatectomy, robotic-assisted laparoscopic prostatectomy

## Abstract

Introduction: Robotic-assisted laparoscopic prostatectomy (RALP) and external beam radiation therapy (EBRT) are two widely recognized clinical standards for treating localized prostate cancer (PCa). This study aims to investigate the prevalence of various post-treatment side effects after RALP, EBRT, and the combination of RALP followed by EBRT.

Methods: This retrospective study utilized a large-scale bioinformatics database through the UF Health Integrated Data Repository, Informatics for Integrating Biology and the Bedside (i2b2 1.7.12a PROD). Males over the age of 35 with PCa who underwent RALP, EBRT, or RALP followed by EBRT were identified. Participants who received other treatments for PCa were excluded. International Classification of Diseases (ICD)-9, ICD-10, and Current Procedural Terminology (CPT) codes were used to identify patients with PCa and post-treatment side effects, while CPT codes were used to isolate those who underwent RALP, EBRT, or RALP followed by EBRT. Patients diagnosed with a given complication before receiving treatment were excluded from the analysis. SPSS was used for all statistical analyses, which consisted of multiple independent chi-square tests. Percentages for each postoperative complication were calculated using the total number of participants who received each treatment.

Results: A total of 2329576 patients were identified from June 2011 to December 2024. Of these, 752491 were males over 35 years old. Among 17729 patients with PCa, 1148 (6.48%) received RALP, 1399 (7.89%) underwent EBRT, and 144 (0.81%) underwent RALP followed by EBRT. The most common side effects in men who underwent RALP were stress incontinence, 690 (60.10%); erectile dysfunction, 567 (49.39%); and gross hematuria, 63 (5.49%). The most common side effects in men who underwent EBRT were erectile dysfunction, 157 (11.22%); gross hematuria, 118 (8.43%); and pelvic pain, 67 (4.79%). The most common side effects in men who underwent RALP followed by EBRT were erectile dysfunction, 142 (98.61%); stress incontinence, 22 (15.28%); and gross hematuria, 21 (14.58%). Gross hematuria, bladder cancer, and prostate-specific antigen (PSA) elevation were significantly more likely in patients who underwent EBRT. Stress incontinence and erectile dysfunction were significantly more common in patients who underwent RALP.

Conclusion: Each treatment modality differs in its postoperative complication profile. Further investigation of these findings is needed to better assess treatment risks and guide physician decision-making for optimal patient outcomes.

## Introduction

Prostate cancer (PCa) is the most common cancer among men globally and a leading cause of cancer-related deaths in the United States [1]. Treatment choices for prostate cancer vary depending on the patient’s age and life expectancy, the disease stage and grade at diagnosis, and overall health status. The primary treatment approaches for localized prostate cancer are radical prostatectomy (RP) and external beam radiation therapy (EBRT) [2]. The integration of robotic assistance, known as robotic-assisted laparoscopic prostatectomy (RALP), has become increasingly popular as a minimally invasive approach to RP for the treatment of PCa [3]. RALP involves the precise removal of the entire prostate gland and seminal vesicles through multiple small incisions in the lower abdomen using robotic devices [4]. Its growing popularity over traditional open surgery is largely due to increasing evidence demonstrating lower rates of postoperative pain, shorter recovery times, and reduced hospital stays [5,6]. Alternatively, EBRT delivers high-energy radiation beams precisely targeted at malignant tumors within the prostate [7]. EBRT is often favored for patients who are poor surgical candidates, typically because of advanced age or comorbidities. EBRT has demonstrated oncologic outcomes comparable to those of RALP while avoiding operative complications associated with general anesthesia and prolonged recovery times [8,9]. RALP is often selected by patients and clinicians seeking definitive local treatment. Understanding the differing complication profiles and prevalence associated with each PCa treatment approach is crucial for patient-physician shared decision-making.

Individuals with prostate cancer face critical decisions regarding their treatment selection. A key factor in procedure selection is how these approaches differ in their side effect profiles. RALP is typically linked to significantly greater risks of stress incontinence and erectile dysfunction compared to EBRT [10]. EBRT is commonly associated with higher rates of fecal incontinence and gross hematuria [11].

Patient treatment selection can be affected not only by side effects, but also by factors such as age, preexisting medical conditions, location of prostate cancer, and personal values. Older patients with preexisting medical conditions typically favor EBRT due to the perioperative complication risks and postoperative functional recovery associated with RALP [12,13]. These risks are particularly salient when considering physiological deterioration due to age and other comorbidities that could increase surgical complications and prevent full recovery. Despite this, technological improvements in minimally invasive surgery have led to promising advances in RALP through heightened surgical precision and reduced complications. These technological refinements seek to reduce operative risks and recovery times, making RALP an increasingly feasible option for older patients with increased risk factors. The objective of this study was to compare the prevalence of coded post-treatment adverse effects among patients with prostate cancer treated with RALP, EBRT, and RALP followed by EBRT within the University of Florida Health system in North Florida.

## Materials and methods

This retrospective study was conducted using aggregate, de-identified electronic health record data obtained through the University of Florida (UF) Health Integrated Data Repository (IDR) and Information for Informatics for Integrating Biology and the Bedside platform (i2b2 1.7.12a PROD) [14,15]. UF i2b2 provides de-identified query outputs from UF Health clinical and research facilities across the state of Florida. No patient-level identifiable data were accessed, exported, or analyzed. Because this study used de-identified aggregate query outputs, it did not involve direct patient contact or review of identifiable private health information. The study was considered exempt from institutional review because it used only de-identified aggregate data. This article was previously presented as a meeting abstract at the 2025 Society of Robotic Surgery Annual Meeting on July 17, 2025.

We identified male patients aged 35 years or older with a diagnosis of PCa who received care at a UF Health facility between June 2011 and February 2024. The underlying PCa cohort included all eligible men with a PCa diagnosis, while the analytic treatment cohort included patients who underwent RALP, EBRT, or RALP followed by EBRT based on the International Classification of Diseases, Ninth and Tenth Revisions (ICD-9 and ICD-10), and Current Procedural Terminology (CPT) billing-linked codes (Table 1) [16-18]. Participants who received other localized PCa treatments, including brachytherapy or alternative prostate procedures, were excluded from the analytic treatment cohort. Post-treatment adverse effects were identified using ICD-9 and ICD-10 diagnosis codes.

**Table 1:** ICD-9, ICD-10, and CPT billing-linked codes RALP: robotic-assisted laparoscopic prostatectomy; EBRT: external beam radiation therapy; PSA: prostate-specific antigen; CPT: Current Procedural Terminology; ICD: International Classification of Diseases; EPIC: Epic Systems Corporation

Codes	CPT	ICD-10	ICD-9
RALP	55866	-	-
EBRT	77280, 77285, 77290, 77261, 77262, 77263, 77295, 77300, 77301, 77321, 77331, 77338, 77306, 77307, 77332, 77333, 77334, 77387, 77385, 77402, 77427, 77431, 77432, 77435, 77469, 77470	-	-
Brachytherapy (excluded)	77750, 77778, 77789, 77790, 77761, 77762, 77770, 77771, 77772, 77316, 77317, 77318, 77327, 77328, 77785, 77786, 77326	-	-
Other surgical procedures on the prostate (excluded)	55801, 55821, 55831, 55810, 55815, 55840, 55842, 55845, 55867, 55870, 55873, 55874, 55880, 55899	-	-
Prostate malignancy	-	C61	185
Urge incontinence	-	N39.41	788.31
Stress incontinence	-	N39.3	788.32
Erectile dysfunction	-	N52	607.84
Gross hematuria	-	R31.0	599.71
Mixed incontinence	-	N39.46	788.33
Pelvic pain	-	R10.2	719.45
Radiation cystitis	-	N30.4	595.82
Fecal incontinence	-	R15	787.6
Bladder malignancy	-	C67	188
Death	Death date (EPIC)		
PSA elevation after treatment	-	R97.21	-
Urethral stricture	-	N99.11	598.2

Data extraction was performed using the i2b2 query tool, which applies Boolean logic (“AND,” “OR,” and “AND NOT”) to define patient cohorts. Each query was constructed using patient demographics, diagnoses, and procedures mapped to their relevant billing codes. Inclusion criteria were combined using “AND” operators to ensure that all required conditions were met (e.g., male sex, age ≥35 years, prostate cancer diagnosis, and treatment type), while “OR” operators were used within categories to capture equivalent codes across coding systems (e.g., ICD-9 and ICD-10 codes for the same diagnosis). Exclusion criteria (e.g., brachytherapy or alternative prostate procedures) were applied using “AND NOT” operators.

Specifically, each treatment event, such as RALP identified by CPT code 55866, was defined as an index event, and adverse effects, such as gross hematuria identified by ICD-10 code R31.0 or ICD-9 code 599.71, were defined as subsequent coded events. Temporal logic was applied so that the first recorded occurrence of treatment preceded the first recorded occurrence of the complication (“Event 1 occurs before Event 2”). For the combined-treatment cohort, queries were structured to identify patients who underwent RALP followed by EBRT in chronological sequence. Because erectile dysfunction could not be reliably temporally attributed within the RALP followed by EBRT cohort using the available query structure, this outcome was interpreted cautiously in that group.

All query components were set as “independent of visit” and required at least one recorded instance of each code within the study period. This approach allowed eligible patients and coded outcomes to be captured across the longitudinal electronic health record, although misclassification of pre-existing, incident, or persistent conditions remains possible in retrospective code-based analyses.

**Figure 1:**
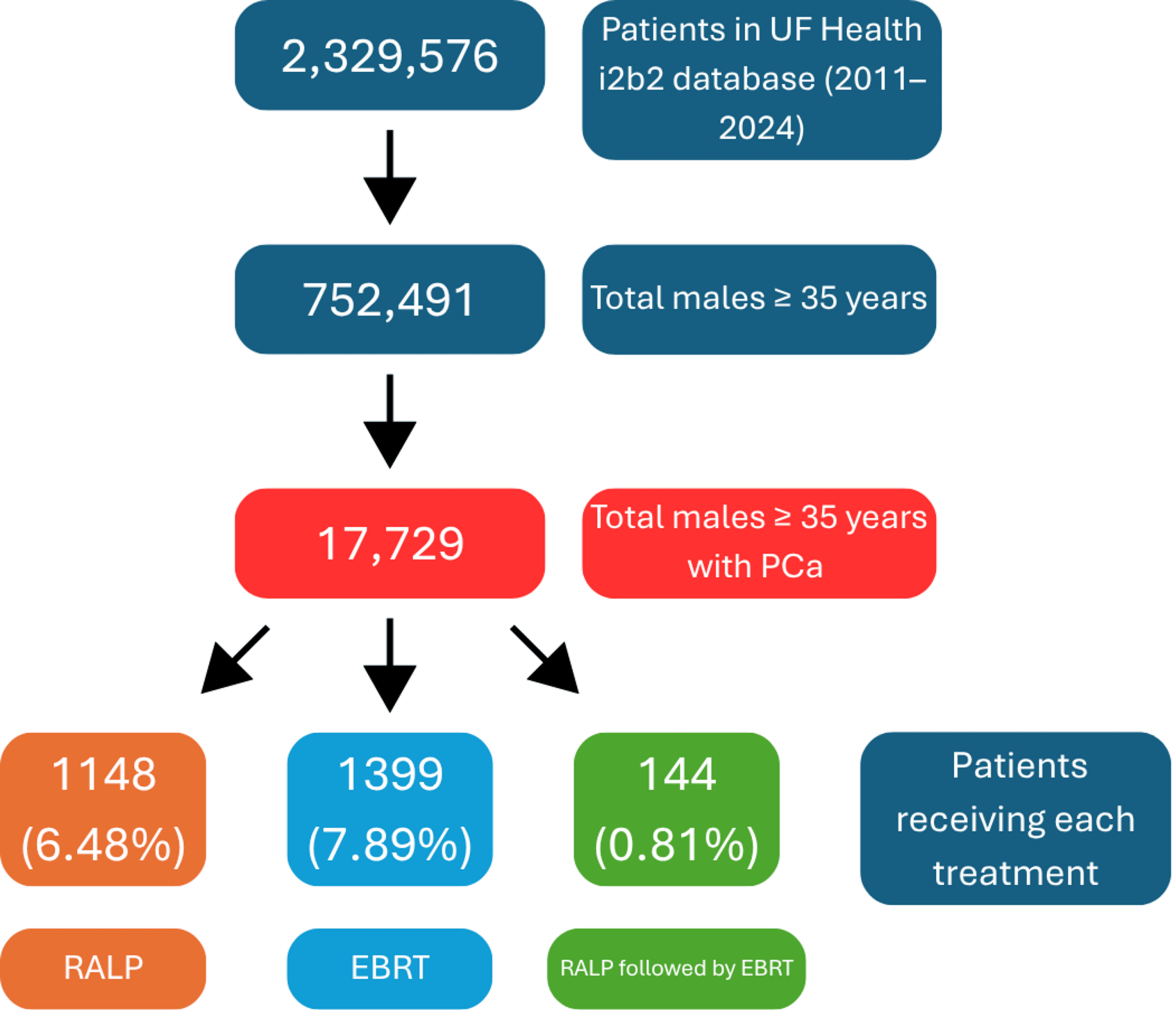
Patients identified using i2b2 RALP: robotic-assisted laparoscopic prostatectomy; EBRT: external beam radiation therapy; PCa: prostate cancer; UF: University of Florida; i2b2: Informatics for Integrating Biology and the Bedside

The entire IDR database included 2329576 patients during the study period. Of those, 752491 were males aged 35 years or older. The underlying prostate cancer cohort included 17729 male patients aged 35 years or older diagnosed with prostate cancer (Figure 1). Among this cohort, 2691 patients underwent one of the three treatment approaches of interest and comprised the analytic treatment cohort.

Statistical analysis was conducted using SPSS version 29.0.2.0 (IBM Corp., Armonk, NY, USA). Descriptive statistics were used to summarize demographic and clinical characteristics. Categorical variables were reported as frequencies and percentages. The prevalence of coded post-treatment adverse effects was calculated for each treatment group: RALP, EBRT, and RALP followed by EBRT. Group comparisons were conducted using chi-square tests. Because i2b2 provides aggregate, de-identified query outputs with privacy-protective masking of reported counts, values should be interpreted as approximate aggregate estimates rather than manually abstracted raw chart counts. Outcomes with counts reported as fewer than three patients were displayed conservatively in descriptive tables but excluded from formal comparative testing when reliable statistical analysis was not feasible. A p-value <0.05 was considered statistically significant.

## Results

**Figure 2:**
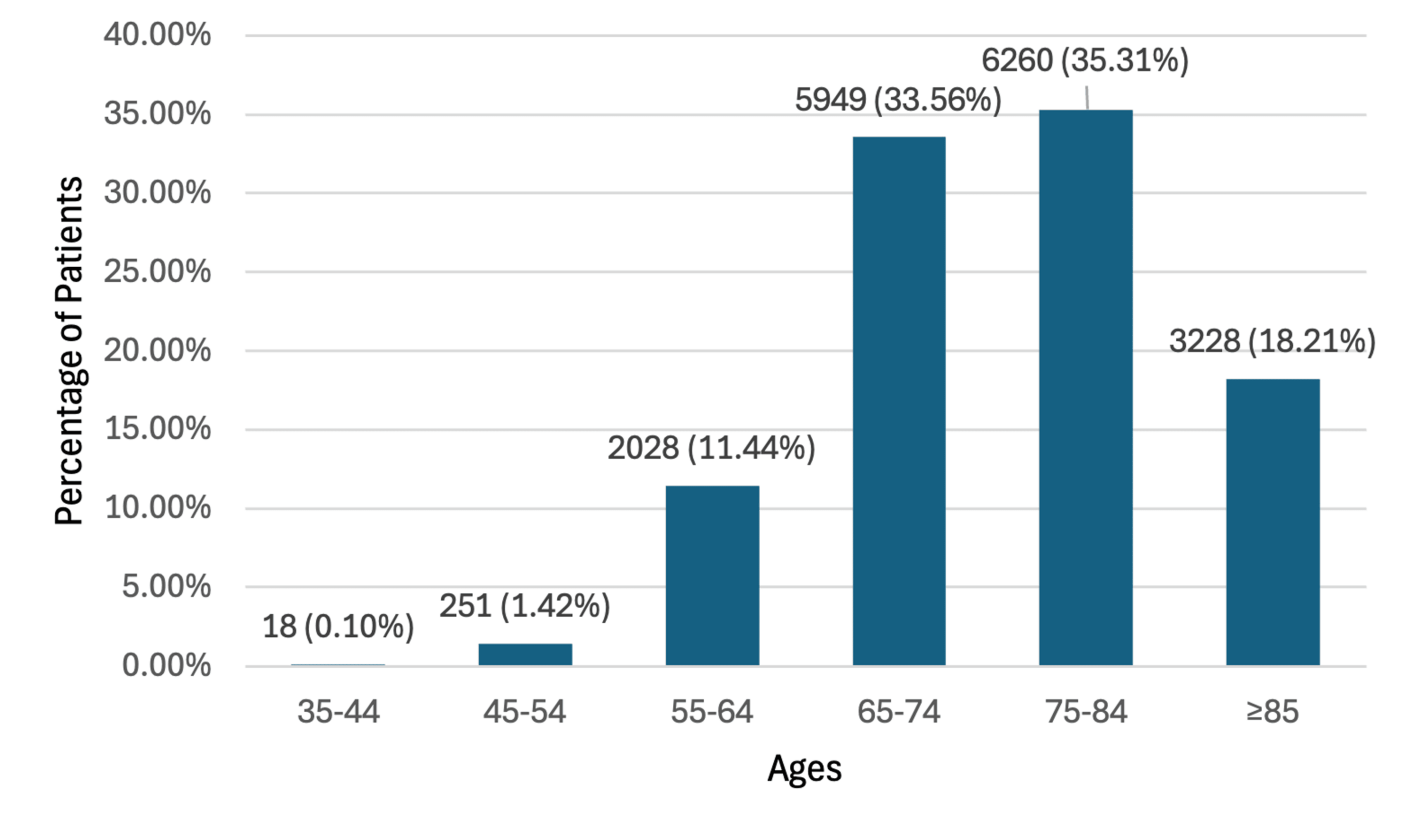
Prevalence of PCa in North Florida by decade of age PCa: prostate cancer

**Figure 3:**
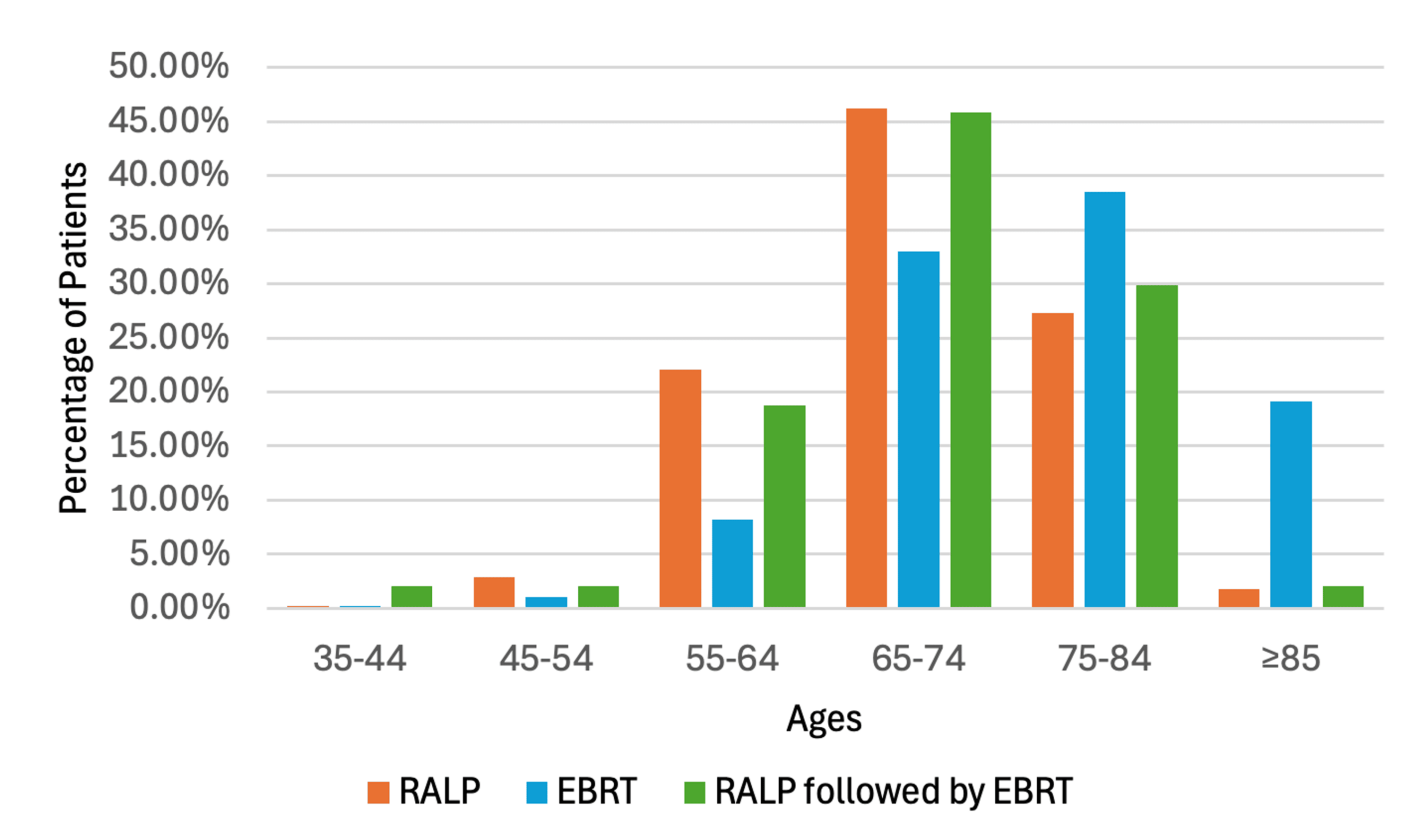
Distribution of treatment type by age group RALP: robotic-assisted laparoscopic prostatectomy; EBRT: external beam radiation therapy

The underlying PCa cohort included 17729 male patients aged 35 years or older. Among these patients, 1148 (6.48%) underwent RALP, 1399 (7.89%) underwent EBRT, and 144 (0.81%) underwent RALP followed by EBRT, comprising an analytic treatment cohort of 2691 patients. The underlying PCa cohort was classified into six age groups: 18 (0.10%) patients aged 35-44 years, 251 (1.42%) aged 45-54 years, 2028 (11.44%) aged 55-64 years, 5949 (33.56%) aged 65-74 years, 6260 (35.31%) aged 75-84 years, and 3228 (18.21%) aged ≥85 years (Figure 2). The distribution of treatment type by age group was also examined (Figure 3). Men treated with RALP had the following age distribution: 35-44 years (0.26%), 45-54 years (2.87%), 55-64 years (22.04%), 65-74 years (46.25%), 75-84 years (27.26%), and ≥85 years (1.74%). Men treated with EBRT had the following age distribution: 35-44 years (0.21%), 45-54 years (1.07%), 55-64 years (8.22%), 65-74 years (33.02%), 75-84 years (38.53%), and ≥85 years (19.09%). Men treated with RALP followed by EBRT had the following age distribution: 35-44 years (2.08%), 45-54 years (2.08%), 55-64 years (18.75%), 65-74 years (45.83%), 75-84 years (29.86%), and ≥85 years (2.08%).

The most prevalent post-treatment adverse effects among participants receiving RALP included stress incontinence, 690 (60.10%); erectile dysfunction, 567 (49.39%); gross hematuria, 63 (5.49%); mixed incontinence, 54 (4.70%); and urge incontinence, 28 (2.44%). The most prevalent post-treatment adverse effects among participants receiving EBRT included erectile dysfunction, 157 (11.22%); gross hematuria, 118 (8.43%); pelvic pain, 67 (4.79%); urge incontinence, 60 (4.29%); and radiation cystitis, 57 (4.07%). The most prevalent post-treatment adverse effects among participants receiving RALP followed by EBRT included erectile dysfunction, 142 (98.61%); stress incontinence, 22 (15.28%); gross hematuria, 21 (14.58%); radiation cystitis, 15 (10.42%); and urge incontinence, 13 (9.03%) (Table 2).

**Table 2:** Prevalence of coded post-treatment adverse effects by treatment type Values represent aggregate i2b2 query outputs and should be interpreted as approximate coded-event estimates. Counts reported as fewer than three by i2b2 were displayed as three for descriptive consistency and excluded from formal comparative testing where appropriate. RALP: robotic-assisted laparoscopic prostatectomy; EBRT: external beam radiation therapy; PSA: prostate-specific antigen; i2b2: Informatics for Integrating Biology and the Bedside

	RALP (N=1148)	EBRT (N=1399)	RALP followed by EBRT (N=144)	P-value
Urge incontinence	28 (2.44%)	60 (4.29%)	13 (9.03%)	<0.001
Stress incontinence	690 (60.10%)	32 (2.29%)	22 (15.28%)	<0.001
Erectile dysfunction	567 (49.39%)	157 (11.22%)	142 (98.61%)	<0.001
Gross hematuria	63 (5.49%)	118 (8.43%)	21 (14.58%)	<0.001
Mixed incontinence	54 (4.70%)	20 (1.43%)	11 (7.64%)	<0.001
Pelvic pain	26 (2.26%)	67 (4.79%)	7 (4.86%)	0.003
Radiation cystitis	-	57 (4.07%)	15 (10.42%)	<0.001
Fecal incontinence	3 (0.26%)	53 (3.79%)	3 (2.08%)	<0.001
Bladder malignancy	11 (0.96%)	36 (2.57%)	3 (2.08%)	0.002
Death	39 (3.40%)	475 (33.95%)	15 (10.42%)	<0.001
PSA elevation after treatment	18 (1.57%)	90 (6.43%)	-	<0.001
Urethral stricture	10 (0.87%)	6 (0.43%)	3 (2.08%)	0.224

## Discussion

Treatment decisions for localized prostate cancer are influenced by physician recommendations, patient preferences, baseline health status, and anticipated quality-of-life outcomes [19,20]. Because RALP and EBRT have distinct functional and adverse-effect profiles, characterizing treatment-associated complications is important for shared decision-making [6]. This retrospective study compared existing data on three treatment options for prostate cancer: RALP, EBRT, and RALP followed by EBRT. The results showed that each treatment type varied significantly in its prevalence of side effects. RALP was associated with significantly higher rates of stress incontinence and erectile dysfunction, whereas EBRT was associated with more prevalent prostate-specific antigen (PSA) elevation, death, fecal incontinence, and gross hematuria. The observed prevalence of each post-treatment adverse effect should be understood as the first recorded occurrence of each complication rather than being defined at a specific follow-up time.

For patients receiving RALP, stress incontinence and erectile dysfunction were significantly more common. Stress incontinence is a frequent concern among post-treatment adverse effects following RALP. Although robotic-assisted surgery is a minimally invasive approach aimed at minimizing lasting damage to the urinary sphincter, such complications have not been eliminated despite advances in robotic technique [21,22]. Stress incontinence was observed in 690 (60.10%) patients in the RALP group and 32 (2.29%) patients in the EBRT group. This finding is consistent with a study by Liu et al., which revealed a higher incidence after RALP compared to radiation therapy [23]. This trend was also observed in the study by Corsini et al., who reported significantly lower risks of urinary incontinence and bother in the radiotherapy group than in the RALP group [24]. A prospective study conducted by Tanji et al. included 299 patients who underwent RALP and found lower rates of severe urinary incontinence, with only 9.0% categorized in this group [25]. Erectile dysfunction was observed at a prevalence of 49.39% in the RALP group and 11.22% in the EBRT group. Other comparative studies consistently support this trend, with robotic-assisted prostatectomy more commonly associated with erectile dysfunction [26,27].

Several coded adverse outcomes were more frequently observed in the EBRT group than in the RALP group, including PSA elevation after treatment, bladder malignancy, death, fecal incontinence, and gross hematuria. PSA elevation after treatment may reflect biochemical recurrence or the need for additional evaluation or treatment, although this code-based outcome could not be clinically characterized in the present study [28].

The prevalence of bladder cancer after EBRT was also higher than that after RALP. The comparison conducted by Monda et al. further supports this finding, as they found higher rates of bladder cancer after radiation compared to RP [29]. Additionally, bladder cancers occurring after radiation tend to be more aggressive, with the study conducted by Suriano et al. demonstrating greater rates of high-grade and muscle-invasive disease compared to the general population [29,30]. They also found that bladder malignancies may be diagnosed later because some clinicians may consider hematuria occurring after radiation to be normal. In the present study, bladder malignancy was observed in 11 RALP patients (0.96%) and 36 EBRT patients (2.57%). Using the same i2b2 query-based methodology, the prevalence of bladder malignancy among all males aged 35 years or older in the broader dataset was 0.82%. Gross hematuria was also more frequently observed among EBRT patients, occurring in 118 EBRT patients (8.43%) compared with 63 RALP patients (5.49%). These findings should be interpreted cautiously because this study relied on coded diagnoses and could not describe timing, etiology, or cancer-specific clinical details.

Fecal incontinence was more frequently observed in the EBRT group than in the RALP group. Putta and Andreyev’s review of existing studies on pelvic radiation therapy reports fecal incontinence as a highly under-recognized side effect, underscoring the need for greater clinical awareness [31]. Hamilton et al. reported similar findings, with a 5.4% decline in bowel function score occurring by the 24-month follow-up period in patients treated with EBRT [32]. Because small cell counts were privacy-protected in the present dataset, low-frequency outcomes should be interpreted cautiously for RALP.

Mortality was identified using the death-date fields available in the electronic health record through i2b2. All-cause mortality was more frequently observed in the EBRT group than in the RALP group, consistent with prior literature showing that treatment outcomes are influenced not only by tumor risk but also by patient age and health status. Ko examined survival rates after robotic-assisted prostatectomy versus radiation therapy in elderly patients with non-metastatic prostate cancer [33]. The study revealed that, when dividing patients using a 75-year age cutoff, the mortality rate was significantly lower in the robotic-assisted prostatectomy group than in the radiation therapy group. In our study, the increased mortality rate in the EBRT group may be largely explained by the fact that 90.64% of these patients were aged 65 years or older. Patients in this age group are generally offered EBRT because of the higher risks associated with comorbid conditions that may preclude surgical intervention [34,35]. These individuals also typically present with more advanced stages of prostate cancer, which could explain the lower survival outcomes observed in this study. However, this trend was not observed in the RALP followed by EBRT group, which had a mortality rate of 10.42%. This may be explained by the significantly smaller sample size of 144 patients compared to more than 1000 patients in the other groups.

One strength of this study is the inclusion of a combined-treatment cohort of patients who underwent RALP followed by EBRT, a group less commonly examined in large retrospective comparisons. Coded erectile dysfunction was highly prevalent in this cohort, suggesting a substantial burden of sexual dysfunction among patients requiring combined local therapy. However, this finding should be interpreted with particular caution because erectile dysfunction could not be reliably temporally attributed within the combined-treatment cohort using the available i2b2 query structure. Therefore, this result should be considered descriptive rather than causal.

There were several limitations to this study. First, the retrospective code-based design relied on previously recorded billing and diagnosis codes rather than clinician-documented outcomes, which introduces the possibility of coding error, undercoding, and misclassification. Absence of a diagnosis code does not necessarily indicate absence of a symptom or complication. Therefore, each reported outcome represents a coded post-treatment event rather than a confirmed causal complication. Second, patients likely differed across groups with respect to age, comorbidity burden, disease severity, surgical candidacy, baseline urinary and sexual function, and other unmeasured clinical factors. Therefore, comparisons between treatment groups should not be interpreted as causal measures of treatment-related risk. For example, raw mortality rates in our study could not be adjusted for age, comorbidities, or disease stage. Third, i2b2 reports aggregate, de-identified, privacy-protected query outputs rather than manually abstracted raw chart data. Reported counts should therefore be interpreted as approximate estimates, and low-frequency outcomes with small or suppressed cell counts should be interpreted cautiously. Fourth, follow-up duration and timing of complication onset were not standardized. Some adverse effects, such as urinary incontinence after surgery, may occur early and improve over time, while others, such as radiation-associated bowel or bladder complications, may arise later. Without standardized follow-up intervals, this study could not distinguish acute, persistent, or late-onset complications. It is also important to note that while 17729 patients were aged 35 years or older and had been diagnosed with PCa, only 2691 patients received one of the three treatment types after diagnosis. Due to the nature of the study design, the treatment course chosen by the remaining patients is unknown, although possibilities include loss to follow-up, active surveillance, or receiving care outside of the UF Health system. At last, details such as radiation dose, disease stage, baseline functional status, surgical technique, surgeon experience, and subsequent salvage therapies were not available through the aggregate query structure, limiting clinical interpretation.

## Conclusions

In this retrospective aggregate database study, our findings highlight significant differences in post-treatment adverse effects across patients treated with RALP, EBRT, and RALP followed by EBRT for prostate cancer. Stress incontinence and erectile dysfunction were more frequently observed after RALP, whereas gross hematuria, fecal incontinence, radiation cystitis, PSA elevation, and all-cause mortality were more frequently observed after EBRT. Understanding the side effects associated with each treatment is necessary to assess risks and guide physician decision-making regarding optimal patient outcomes. These findings support the need for individualized, patient-centered shared decision-making, but they should be interpreted in the context of important limitations, including non-random treatment selection, variable follow-up, privacy-protected aggregate counts, and reliance on diagnosis coding.

Future prospective studies with standardized follow-up intervals, patient-reported outcomes, and adjustment for baseline clinical differences are needed to better characterize comparative adverse-effect profiles across prostate cancer treatment modalities. Further research may improve shared decision-making and physicians’ ability to counsel patients on the long-term implications of each treatment modality.
